# Discussions of the Indiana State Dental Association

**Published:** 1860-02

**Authors:** 


On motion adjourned.

S. B. Smith, *Secretary.*

### DISCUSSIONS OF THE INDIANA STATE DENTAL ASSOCIATION.

1. Should pivot teeth be inserted, if so, under what circumstances, and in what manner?

Dr. Ulrey thinks it useless to talk much on the subject. Only under the most favorable circumstances is the dentist justified in inserting teeth on pivot. Relates a case in which pivot teeth were worn with satisfaction for twenty-one years, on wood pivots. His general experience is, that they may be worn from one to three years; and in very favorable cases from six to seven years. As to the manner, some use metallic pivots, others plain wood, others wood with foil or silk, can hardly say which is best, but would prefer a plain hickory pivot as it is less liable to split the tooth by a sudden jar. Thinks it good practice to fill the canal of the fang beyond the pivot.

Dr. MOORE thinks it makes a very durable, serviceable, and in appearance natural, dental substitute. Prefers to have patients with thick, strong jaws. There is generally not sufficient attention given to pivot work. Perfect adaptation is required. In good roots and favorable cases, if well inserted, pivot teeth are always satisfactory.

Dr. MANLOVE is in the habit of filling the fang above the pivot with asbestos or tin foil; finds work of this kind to last from three to four years; they do better with wooden than metal pivots. Has one case in which the fang was ulcerated; treated it in this manner, and inserted a pivot tooth, seven years ago, and it is still in a good condition.

Dr. SMITH starts out with the proposition never to insert a pivot tooth when he can avoid it. Performs the operation occasionally through the importunities of the patient; finds inflammation and ulceration almost universally supervene; and then if he can possibly get the consent of the patient, extracts the fang, and mounts the tooth on plate. Uses hickory wood pivots, well prepared, drills out the canal and fills to the point of the fang.

Dr. LUPTON has had some experience, in most cases very favorable. Do not like to insert on unhealthy fangs, but would, like Dr. Smith, prefer in such cases, to extract and insert on plate; has quite a number of cases, inserted several years ago, that are still doing well.

Dr. HUNT does mount teeth on pivot. In best operations fills to the end of the fang; drills out the fang and fills and inserts a metallic tube, fills around it with adhesive foil, and attaches the teeth with gold pivots; by this method the teeth are kept more cleanly, and are more satisfactory than by any other method. Hollows out the orifice of the canal, and fills with gold, so as to expose as little of the dentine at the articulating surface of the fang as possible. Does in some instances, insert in the old way on wood pivots; but does not consider it good practice without first treating the fang. Is decidedly in favor of pivot teeth. The fulness of arch, and expression of face and features are preserved, as they can be in no other way; has had no trouble from inflammation or ulceration in any of the cases thus prepared. Drills out the fang, then places the tube and has an assistant to hold it in position while filling around it. Grinds off the inside of the artificial crown, to open the hole, then with a small gold pivot, kept in place with wax, place the tooth accurately in its position in the mouth, then remove carefully and solder.

Considers it the duty of the dentist to preserve the form and features of the face as long as possible.

Dr. JOHNSTON agrees with Dr. Hunt. He inserts pivot teeth and preserves the natural roots as long as possible, because it more perfectly conceals the operation; should always fill the fang above the pivot with gold. When the teeth are diseased they should be treated till they are restored to a healthy condition; and in such cases finds no more difficulty than from filling a tooth, the nerve of which has been exposed.

Thinks he has made a discovery which will entirely revolutionize pivot work, both in the manufacture of the teeth, and in the mode of insertion; but as the plan is not yet perfected, does not at present feel at liberty to make it public, for fear of an accident. *Does not intend to patent.*

Dr. HARLAN, practice similar to that of Drs. Hunt and Johnston, except that he never attempts to treat an unhealthy root, prefers to put in a plate when the patient will stand the expense.

Dr. STANLEY came to listen, not to talk, being young in the profession. Generally finds the root in an unhealthy condition, then recommends extraction, though does insert occasionally, then uses seasoned hickory pivots.

Dr. MORRIS on healthy roots, and favorable circumstances, performs the operation; fills the fang above the pivot with gold; uses hickory wood for pivots. Refuses diseased roots.

Dr. TURNER has had but little experience in this operation; his knowledge is derived mostly from observation. Remembers a case in which a pivot tooth was worn for twenty years. Re-set one eighteen months ago which had been worn eight years.

Dr. PERINE has not inserted a pivot tooth for years: he relates the method of Dr. Clark, of New Orleans, as described by Dr. Hunt, viz: to drill out the fang and fill with gold, then drill a hole through the gold and mount the tooth on a gold pivot, which enables the patient to remove and cleanse it; there are many methods of performing this operation; and much good work has been done in the old way, on hickory pivots; the roots being healthy and the fang filled above the pivot.

Dr. KNAPP thinks the ground has been thoroughly canvassed, it is well now to let the subject rest. If he does say any thing fears he will produce some hearsay. If he could get patients to pay for it would adopt the mode described by Dr. Hunt as mere fancy, would beg leave to adopt it; but we must take cases as they present themselves. We generally find those desiring pivot teeth, expect them to last only a few years. His method is to drill out the decay, and fill to the end of the fang with Townsend's Amalgam, and while this is plastic, force the tooth and pivot into the proper position; by this method they will last three or four years.

Dr. PERINE related a case, when upon examination, it was proposed to separate the central incisor, but was informed by the patient that they were artificial teeth, and on close inspection, found they were human teeth inserted on pivot; and so nicely adjusted as to almost defy detection.

Adjourned to 1½ o'clock, P. M.

### AFTERNOON SESSION.

### SECOND SUBJECT—*Treatment of Inflamed Dentine.*

Dr. ULREY we are frequently called upon to treat this affection. When a case is presented, and on application of an instrument, find it to give pain, concludes it is inflamed dentine. If he can have control of the patient, treat with a view of correcting a vitiated condition of the secretions. If this can not be done use escharotics; these do not restore to health but destroy the diseased part. When chlorid of zinc will not accomplish the object, uses cobalt with creosote. This is dangerous treatment, but we all sometimes resort to it. (*A few of us do not.* ED.) The best thing is to keep the cavity filled with creosote and cotton, from day to day, until the inflammation abates, but when there is not time for this use cobalt, but unfortunately this accomplishes more than is desired.

Prefers chlorid of zinc and creosote, yet use cobalt, which is in reality crude arsenic.

For sensitiveness about the necks of the teeth, touches with chlorid of zinc; this destroys the sensation, so that when the part is touched with an instrument, there is no pain experienced.

Dr. JOHNSTON does not think the subject will admit of much discussion; overcomes the difficulty by the application of chlorid of zinc, applied in as dry a state as possible. After deliquescence the material becomes worthless. In the first place excavate the cavity as thoroughly as possible; apply several times in some cases. As soon as it is dissolved the force is spent; dries out the cavity and reapplies, which soon subdues the pain; although on first application it produces a sharp, stinging pain, similar to toothache; which, however, lasts only a few moments.

When the chlorid does not answer uses a very sharp instrument in preference to using cobalt or arsenic.

Dr. Lupton has had some experience in the use of chlorid of zinc, but generally found the application to produce about as much pain as the tooth-ache itself. Has also used cobalt, and with great satisfaction; there being no pain or sensation. In one case there was a small opening into the pulp, and found it had gone farther than bargained for.

Dr. Knapp uses arsenic and creosote more frequently than any thing else, but rarely permits the patient to leave the office with it in the tooth, and has yet to see the first case in which injury has resulted. Permit it to remain in the tooth from five minutes to half an hour.

Third Subject—*Treatment of Dental Periostitis and Alveolar Abscess.*

Dr. Dills yesterday morning filled a lateral incisor which was dead,—in course of a day or two the patient returned to have the work doue; there were symptoms of periostitis, produced counter irritation by the use of chloroform and sulph ether, and directed an occasional dose of pills.

Dr. Johnston in the treatment of abscess passes an instrument through the point of the fang, so as to facilitate the discharge of pus through the nerve canal, leaves it so, for a while, then enlarges the canal and passes up chlorid of zinc, or creosote; prefers the former, as it is a better dissinfectant; with the two generally succeeds without further difficulty.

There are cases in which the disease returns, but generally the cure is permanent. Continues treatment thoroughly until the parts are perfectly healthy, to do this it is necessary to have entire command of the time and attention of the patient.

Dr. Manlove agrees with Dr. Johnston, except that he uses in addition tincture of myrrh and tannin. In ordinary cases require about ten days to treat a case. In difficult cases uses creosote right to the point.

Dr. Hunt has nothing new, except the use of leeches, uses them freely in periostitis, and thinks he has saved may cases of periostitis from resulting in alveolar abscess; when lancing or scarifying would not have served the purpose.

Dr. Ulrey admits that the application of leeches is the very best treatment which can be adopted, but not having access to them, uses scarification, and applies chloroform, ether and camphor, and other similar applications. If a greater sore can be made outside than exists inside, it may do some good. Directs also internal remedies, such as purgatives, &c., but when abscess is formed, finds extracting the only certain remedy. Do not see the propriety of keeping a tooth in the mouth that requires constant coaxing. Mentioned the case of a gentleman who wore a pivot tooth, it caused great pain; removed the pivot and introduced a broach, there was an offensive odor, introduced the broach again, and used it as a pump, made an opening for pus to escape, and thus relieved the difficulty.

Periostitis generally results in abscess and death of the tooth. The best way is at once to remove the offending member.

Dr. Moore—a case; patient twelve years old; tooth dead, thick layer of dentine on the pulp chamber, hesitated as to the best course to pursue, but filled the tooth without pain or inconvenience; a few days after was sent for, called and found the patient in a critical condition, with abscess threatening to open outside; after some urging prevailed upon the patient to permit lancing, which gave relief after the inflammation had subsided, found the case just as when filled.

Dr. Johnston supposes that there was an opening into the pulp chamber which the filling closed, and prevented the escape of pus and vitiated matter. If the tooth was dead, should have drilled through to the pulp chamber, treated, and then filled.

Dr. KNAPP suggested that the violence of an operation will sometimes produce inflammation.

Dr. DILLS stated that when the case already described, was presented, he was not certain that the nerve was dead, did try to drill into it, but found the walls so thick it was abandoned, the tooth ached both before and after filling, am now satisfied that the tooth was either dead or a process of ossification of the pulp was in progress.

Dr. KNAPP. If the patient will, in the early stages of periostitis, take a strong emetic it will generally give relief. A case of alveolar abscess, treated six weeks, there was a fistulous opening, with a drill made a free opening, and treated with creosote and chlorid of zinc as before described; cut away the process with a curved lancet, and introduced pledgets of cotton moistened with creosote; in a month the discharge ceased, and a month after the tooth was filled with "Hill's Stopping;" it has given no trouble since; if it continues so will soon fill with gold.

Dr. ULREY thinks it would be profitable to inquire into the *cause* of periostitis, as well as its treatment. He frequently finds difficulty in an *apparently* healthy tooth: in a tooth in his own mouth there are occasionally symptoms of periostitus, which he thinks are produced by exostosis.

Dr. JOHNSTON gave a case in which there was great pain, that could not be accounted for, though from biting on some hard substance the pain increased, and swelling ensued; applied leeches without effect; continued to swell, applied anodynes, iodine, ticture arnica, &c., but in spite of all the efforts the swelling increased, until it forced an opening, with a great discharge of pus; after discharging for a considerable length of time, the opening closed, and a healthy condition was restored.

Dr. MANLOVE desires to known what is to be done when teeth are worn down so near the nerve as to become sensitive and painful?

Dr. HUNT. A case of a tooth quite loose, and discharging, so much so, you could feel the pus fluctuating under the finger; the end of the fang and part of the process absorbed. After the examination commenced the treatment, and continued four months, and finally had the satisfaction of restoring it to a perfectly healthy condition, filled it and it has since done well; the patient values the tooth highly and has since declared it to be more comfortable than for twenty years. He inquired what would be a successful case of fang filling? If it swells and gives pain once is it considered successful? or must it be entirely without pain to be considered successful? Referred to a case filled five years ago, and it swelled and suppurated several years after; it is now healthy and useful.

FOURTH SUBJECT.—*Mechanical Dentistry, including all the various modes of constructing Artificial Dentines.*

Dr. MOORE scarely knows what to say, there are so many good ways and so many bad ways, all being good if well done. In his own practice has adopted continuous gum work, and vulcanized rubber, thinks the latter will not absorb secretions.

Dr. DILLS exhibited a neat specimen of sectional vulcanite work in his own mouth. He spoke of its advantages and disadvantages; thinks it will not absorb the secretions of the mouth; can say but little, from his own experience, having worn it but a few days.

If after it is vulcanized it does not fit, put it into hot water, to soften it, then put it on the cast and adapt it by pressure, thus making a perfect fit; this might be an objection in sectional pieces, as in some cases a sufficient amount of substance can not be supplied to give the required strength and stiffness, when the connections are very small.

Some beautiful specimens of "Amber Base" and Vulcanite were here exhibited which elicited some interrogatories, answers and comments.

Adjourned to 7 o'clock, P. M.

EVENING SESSION.

Association met at 7 o'clock, P. M.

On motion of Dr. Ulrey,

*Resolved*, that the subject of fees and a fee bill be now taken up for consideration.

The subject was ably discussed and the different points presented by Drs. Lupton, Johnston, Dills, Ulrey, Knapp and Stanley.

Dr. HUNT remarked that if a fee bill was adopted it should be in no particular below the one recommended at the last meeting; if any variation make it higher; and that it be obligatory on members to charge no less, but as much higher as they choose.

On motion of Dr. Johnston,

*Resolved*, that the Indiana State Dental Association recommend the following fee bill to the profession of Indiana as a proper minimum of prices for the various operations therein named; and urged members as far as possible to adopt and adhere to it.

For FEE BILL, see page 363, present number.

By request Dr. Moore read an able essay on the duties of the members of the Association. Dr. Moore was requested to furnish a copy of the address for publication.

Dr. KNAPP suggested that if members would write out short articles on topics not embraced in the subjects for discussion it would be attended with great benefit.

On motion of Dr. Johnston,

*Resolved*, that we omit subjects five and six, and proceed to miscellaneous and unfinished business.

SEVENTH SUBJECT.—*Miscellaneous and unfinished Business.*

Dr. LUPTON exhibited some casts and appliances for the correction of irregularity, with a description of the method of using the latter. (Which will be published in the next number of the *Register*. Ed.)

The minutes were read, corrected and adopted. After which the Association adjourned.

